# Expression and evolutionary divergence of the non-conventional olfactory receptor in four species of fig wasp associated with one species of fig

**DOI:** 10.1186/1471-2148-9-43

**Published:** 2009-02-20

**Authors:** Bin Lu, Nina Wang, Jinhua Xiao, Yongyu Xu, Robert W Murphy, Dawei Huang

**Affiliations:** 1 College of Plant Protection, Shandong Agricultural University, Tai'an, Shandong 271018, PR China; 2 Institute of Zoology, Chinese Academy of Sciences, Beijing 100101, PR China; 3 Chengdu Institute of Biology, Chinese Academy of Sciences, Chengdu, Sichuan 610041, PR China; 4 Graduate School of the Chinese Academy of Sciences, Beijing 100049, PR China; 5 Department of Natural History, Royal Ontario Museum, 100 Queen's Park, Toronto, ON M5S 2C6, Canada; 6 State Key Laboratory of Genetic Resources and Evolution, Kunming Institute of Zoology, the Chinese Academy of Sciences, Kunming 650223, PR China

## Abstract

**Background:**

The interactions of fig wasps and their host figs provide a model for investigating co-evolution. Fig wasps have specialized morphological characters and lifestyles thought to be adaptations to living in the fig's syconium. Although these aspects of natural history are well documented, the genetic mechanism(s) underlying these changes remain(s) unknown. Fig wasp olfaction is the key to host-specificity. The *Or83b* gene class, an unusual member of olfactory receptor family, plays a critical role in enabling the function of conventional olfactory receptors. Four *Or83b* orthologous genes from one pollinator (PFW) (*Ceratosolen solmsi*) and three non-pollinator fig wasps (NPFWs) (*Apocrypta bakeri, Philotrypesis pilosa *and *Philotrypesis *sp.) associated with one species of fig (*Ficus hispida*) can be used to better understand the molecular mechanism underlying the fig wasp's adaptation to its host. We made a comparison of spatial tissue-specific expression patterns and substitution rates of one orthologous gene in these fig wasps and sought evidence for selection pressures.

**Results:**

A newly identified *Or83b *orthologous gene was named *Or2*. Expressions of *Or2 *were restricted to the heads of all wingless male fig wasps, which usually live in the dark cavity of a fig throughout their life cycle. However, expressions were widely detected in the antennae, legs and abdomens of all female fig wasps that fly from one fig to another for oviposition, and secondarily pollination. Weak expression was also observed in the thorax of PFWs. Compared with NPFWs, the *Or2 *gene in *C. solmsi *had an elevated rate of substitutions and lower codon usage. Analyses using Tajima's *D*, Fu and Li's D* and F* tests indicated a non-neutral pattern of nucleotide variation in all fig wasps. Unlike in NPFWs, this non-neutral pattern was also observed for synonymous sites of *Or2 *within PFWs.

**Conclusion:**

The sex- and species-specific expression patterns of *Or2 *genes detected beyond the known primary olfactory tissues indicates the location of cryptic olfactory inputs. The specialized ecological niche of these wasps explains the unique habits and adaptive evolution of *Or2 *genes. The *Or2 *gene in *C. solmsi *is evolving very rapidly. Negative deviation from the neutral model of evolution reflects possible selection pressures acting on *Or2 *sequences of fig wasp, particularly on PFWs who are more host-specific to figs.

## Background

The interactions of flowers and insect pollinators are the classic examples of co-evolution. The intimate relationships and specialization between figs and fig wasps are among the best studied cases [1-3]. Pollinating fig wasps (PFWs) usually exclusively pollinate species of figs [2,4-7], although recent discoveries indicate that the species-specific association is much less specific than has been thought previously [8-13]. The pollinators completely depend on figs for their life cycles [14-17]. In addition to PFWs, several non-pollinating species of fig wasps (NPFWs) also exploit figs [18]. Evidently, non-pollinators are less specific to a given host species than pollinators [13,18-23].

The host-specificity behaviour of insects relies heavily on olfaction [24], which has evolved to a level of extreme sensitivity and specificity [25]. This highly discriminative sensory mode is facilitated by an odour-activated seven-transmembrane-domain G protein-coupled receptor (GPCR) that signals cascades [26]. Fig wasps are no exception. The mechanism underlying the host-specificity of fig wasps is almost certainly based on volatile chemicals released by the fig [27-29]. Fig wasps should be able to distinguish the particular odours emitted by hosts from other volatile compounds [30-32]. Different species of wasps appear to have unique volatile profiles which could account for host-specificity [28]. Such chemical constraints likely reduce host switching. Currently, investigations on adaptive changes in fig wasps are limited to morphological characters associated with an ecological niche [14,33-36]. For example, a pollinator must enter the syconium to deposit eggs. Thus, their antennae are easily broken to ease crawling through the ostiole. Unlike PFWs, most NPFWs cannot enter syconia. They usually possess a long ovipositor that is inserted through the syconial wall for egg laying [37-39]. Furthermore, both eyes and wings in most adult male PFWs exhibit highly vestigial traits, a correlate of living in the dark fig cavity all their life [40,41]. Unlike these attributes, very little is known about genetic changes in fig wasp's olfactory system.

As members of the GPCR superfamily, olfactory receptors (ORs) for odorous compounds play the critical role in the olfactory process [42]. The process consists of several linked systems ranging from stereo chemical recognition to the generation of an odour code in the brain. A characteristic trait of conventional odour ligand-binding OR types is the tremendous diversity of their sequences, often exhibiting only ~20% identities to each other [43-45]. A highly conserved, non-conventional member in the insect OR family is known as *Or83b *[46]. Orthologs have been identified from *Drosophila melanogaster *(*DOr83b*) [47-49], *Anopheles gambiae *(*AgOr7*) [50], *Heliothis virescens *(*HvirR2*) [51], *Apis mellifera *(*AmelR2*) [52] and others [50,53,54]. Apparently, *Or83b *does not directly respond to odorants but rather acts as a chaperone receptor to form heterodimers with other odorant and pheromone receptors, thus ensuring dendritic localization [55-57].

While spatial expression patterns of conventional ORs are restricted in small subpopulations of olfactory sensory neurons (OSNs), *Or83b *is co-expressed with conventional ORs in most, if not all, neurons [52,55]. Tissue-specific expression patterns of putative ORs have been observed in ecologically distinct species. For example, in the hematophagous mosquitoes *An. gambiae *and *Aedes aegypti*, *Or83b *orthologs are expressed in the antennae, legs and proboscis (i.e., general gustatory organs). As such, *Or83b *orthologs may be involved in locating human hosts for blood feeding [50]. Sex-specific expression patterns occur in both mosquitoes and moths, for blood ingestion and mate searching, respectively [58-60]. Insects employ ORs to recognize and discriminate various quantitative or special odours in their ecological niche. Therefore, host specialization could reflect selection acting on ORs [61,62]. For example, bee-specific rapid expansion of the OR family presumably underlies their remarkable olfactory abilities, including perception of several pheromone blends, kin recognition signals, and diverse floral odours [45]. In *Drosophila sechellia*, rapid evolution and lack-of-function mutations in olfactory and gustatory receptor genes following a host shift reflect positive selection and/or relaxed constraints associated with an altered ecological niche [62].

In view of prior studies in mosquitoes and moths, different tissue-specific expression patterns of *Or83b *orthologs are expected to occur between resident male and host-searching female fig wasps, or between PFWs that deposit eggs within the figs, and NPFWs that do not. If the tissue-specific expression patterns occur together with the crucial role of *Or83b *orthologs in locating a host, we can infer selection pressure on *Or83b *orthologous genes. The different degree of host specificity between pollinator and non-pollinators [13,18-23] likely makes *Or83b *orthologous genes subject to different magnitudes of selection. Our experimental group consists of one species of PFW (*Ceratosolen solmsi*) and three species of NPFWs (*Apocrypta bakeri*, *Philotrypesis pilosa* and *Philotrypesis* sp.), all associated with *Ficus hispida*. Herein, we identify *Or83b *orthologous genes in these four species of fig wasps, compare spatial expression patterns and substitution rate of these genes in fig wasps, examine possible evolutionary forces, and explore molecular mechanisms involved in co-evolution.

## Results

### cDNA cloning

The newly identified genes of *C.solmsi*, *A.bakeri*, *P. pilosa *and *P*. sp. were named as *CsmOr2, AbOr2, PpOr2 *and *PsOr2*, respectively, and the sequences were deposited in GenBank [Accession numbers: EU281848, EU281849, EU281850, EU281851]. The coding regions of these data had the same length in all fig wasp species (1422 bp when the stop codon was not considered). The length was same as in *Nasonia vitripennis*, but differed from *Microplitis mediator *by a one-codon indel (3 bp) and from *Ap. mellifera *by two indels. The amino acid (aa) sequences were extraordinarily conservative among our fig wasps, with > 90.3% identity (the percentage of identical matches between the two sequences over the reported aligned region) and > 96.4% similarity (the percentage of matches between the two sequences over the reported aligned region where the scoring matrix value is greater or equal to 0.0). This suggested that the four species of fig wasp are closely related relative to other species. Sequence identities ranged from 75.3% to 91.4% when compared with other hymenopteran orthologs including *N. vitripennis*, *M. mediator *and *Ap. mellifera*. Alignment of this non-conventional receptor and the other insects showed that the 19 protein sequences shared greater than 60% identity and 72% similarity. Remarkably, extreme conservation was discovered in the final 164 aa of the C-terminal, where the 19 protein sequences shared nearly 90% identity.

**Figure 1:**
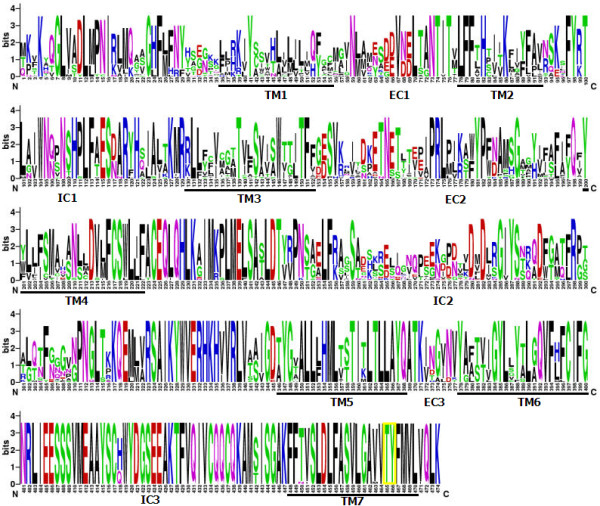
**Alignment of *Or83b *orthologous amino acids in insects**. Conservation of amino acid sequence is displayed as a sequence logo. The relative frequency with which an amino acid appears at a given position is reflected by the height of its one-letter amino acid code, with the total height at a given position proportional to the level of sequence conservation. Transmembrane domains (TM 1–7) and extracellular (EC) and intracellular domains (IC) are numbered and indicated. Threonine (T) and tyrosine (Y) residues that are sites of potential phosphorylation are enclosed in heavy yellow boxes.

We discovered the typical membrane topological structure of insect ORs in the four fig wasp *Or2 *protein sequences for seven putative transmembrane (TM) domains (Figure 1). The N-terminal of these receptors, located intracellularly [56,63], occurred in all protein sequences. The putative membrane spanning domains were inferred to occur at similar relative positions in *CsmOr2, AbOr2, PpOr2 *and *PsOr2*. Remarkably, the N-terminal between TM 1 and TM 2, and from TM 3 towards TM 4, contained the most variable regions. The variable second intracellular loop (IC2) connecting TM 4 and TM 5 was much longer than the other five loops.

### Phylogeny of the *Or2 *genes

**Figure 2:**
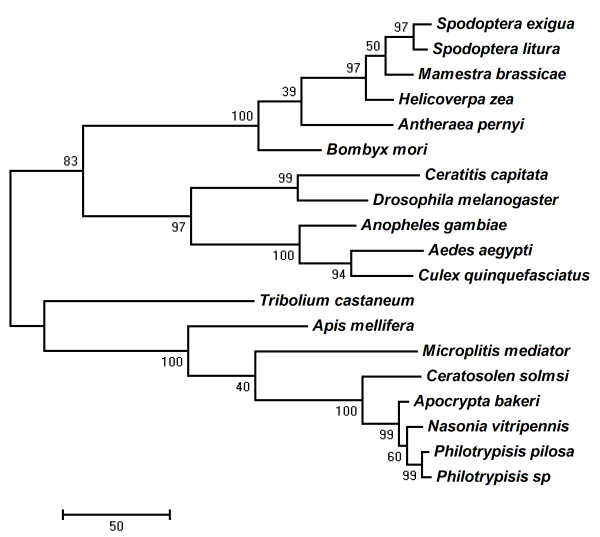
**The maximum parsimony tree of *Or2 *genes from fig wasps**. The MP phylogram was constructed based on 1st + 2nd codon positions. The reliability of each tree node was assessed by bootstrap proportions with 1000 replications. Branch lengths are proportional to change.

The phylogenetic analyses using 1st and 2nd codon positions showed that four fig wasp plus *N. vitripennis Or2 *genes formed a well-supported group (Figure 2; 100% bootstrap support). A sister group relationship of *Or2 *genes was detected for *P. pilosa *and *P*. sp. (99% bootstrap support). The sequence of *N. vitripennis *was more closely related to *Or2 *genes of *P. pilosa *and *P*. sp. than to *A. bakeri*, although this association was weakly supported (60% bootstrap). The *Or2 *gene of the pollinator was dissimilar to those of the three NPFWs. The hymenopterans *Ap. mellifera *and *M. mediator *clustered with the fig wasps plus *N. vitripennis *(100% bootstrap support). All dipteran and lepidopteron sequences formed strongly supported groups (97% and 100%, respectively). Removal of the sequence of *Ceratitis capitata *from the analysis did not affect tree structure. Thus, the sequence was retained in subsequent analyses. Use of aa sequences instead of 1st and 2nd codon positions yielded a nearly identical tree topology except that *N. vitripennis *first clustered with *A. bakeri *(47% bootstrap support) and then formed a group with *P. pilosa *and *P*. sp. (98% bootstrap support) (tree not show). When we used a Sankoff (step matrix) in PAUP* [64] to force the minimum number of mutations (steps) required to transform from one aa to another, we obtained the same trees found without using the matrix (tree not show). The difference in tree topologies likely resulted from a difference in potentially parsimony informative characters between the aa data (210 sites) and 1st and 2nd nucleotide codon positions (355 sites).

### Tissue specificity of expression

**Figure 3:**
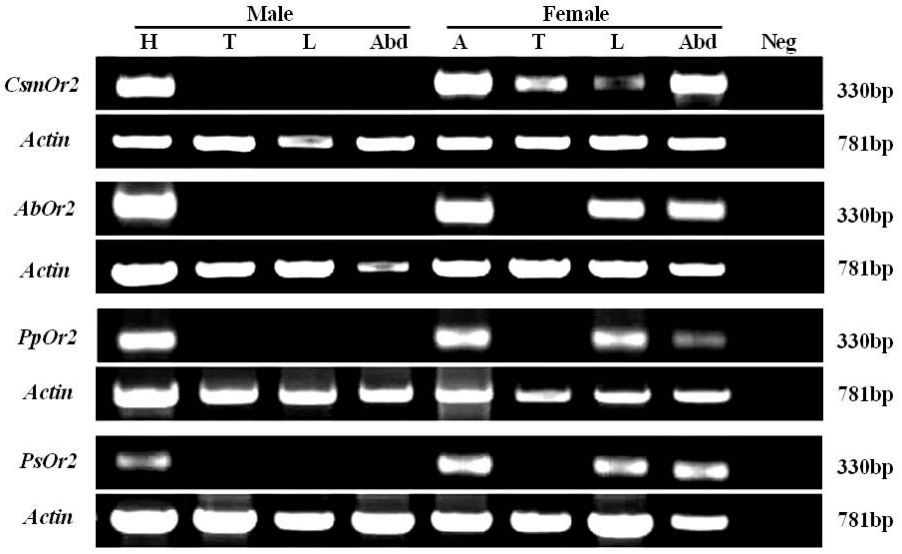
**Olfactory tissue-specific expression of *Or2 *genes in fig wasps**. RT-PCR was performed with cDNAs prepared from different species and tissues of fig wasp. Reaction products were visualized by ethidium bromide staining and UV illumination. Bands were the expected size based on the primer design. A no-template negative control ensured the specificity of the amplification. *Actin *was amplified from each sample as a control for RNA integrity. *CsmOr2*, *Ceratosolen solmsi Or2*; *AbOr2*, *Apocrypta bakeri Or2*; *PpOr2*, *Philotrypesis pilosa Or2*; *PsOr2*, *Philotrypesis *sp.* Or2*; A, antenna; T, thorax; L, leg; Abd, abdomen; H, head; Neg, negative control.

Following PCR for tissue specific-expression (Figure 3), all bands were the size expected from the primer design (330 bp). Bands of similar intensities obtained with primers specific to the *actin *control indicated the integrity of the cDNA preparations. The intensity of the products (relative to an internal control) indicated variable levels of expression in different tissues. Strong expressions of this OR-type occurred in male heads (including antennae and maxillary palps) and in female antennae, in PFW and all types of NPFW. Lower levels of expression were discovered in the abdomens and legs of females in all species of fig wasp. In contrast to female NPFWs, a band of lower intensity was also obtained with cDNA from the thorax of female *C. solmsi*. No transcripts were detected within non-olfactory tissues (e.g., thorax, abdomen and legs) of all males. In all cases, *actin *amplifications were more robust for tissue templates, reflecting higher template quantities in the parallel control reactions. This further demonstrated the absence of detectable expression of ORs in non-olfactory tissues. Finally, to confirm this pattern, an additional 10 cycles of PCR were added to the protocols. Even under these extremely sensitive conditions, RT-PCR products were not detected in all male non-olfactory tissues and in female NPFW thoraxes. Genomic contamination of cDNA templates was clearly distinguishable from cDNA products by primers that spanned predicted introns (data not shown). To verify their specificity, the RT-PCR products from each tissue cDNA were sequenced, revealing that a specific product had indeed been obtained in each instance.

### Selection pressure on individual amino acid sites

Because adaptive evolution usually occurs on a small region of a gene's sequence and can even be restricted to a single aa site [65-67], we sought aa sites that were subjected to selection pressure. Selection pressure can be inferred from the ratio of nonsynonymous to synonymous changes, *Ka*/*Ks *(also known as *d*_*N*_*/d*_*S *_or ω). When *Ka*/*Ks *= 1, neutral selection is indicated and when *Ka*/*Ks *> 1, positive selection is implied. Alternatively, when *Ka*/*Ks *< 1, purifying selection is suggested [68]. In the present study, two thresholds for significance (0.1 and 0.2) were taken into account in order to identify residues potentially involved in ligand-binding activities. In our analysis, *Ka*/*Ks *= 0.0844161; no positively selected nucleotide sites were detected at the *P *< 0.1 in the SLAC analysis. However, 380 of 474 aa sites were negatively selected. *Or2*-type receptors appeared to be primarily negatively selected. When significance level was adjusted to *P *< 0.2, one positively selected site was discovered at the 21st aa (*P *= 0.157371). Serine (S) occurred at this position in the PFWs, but all NPFWs had Glycine (G). The other species of insect displayed various amino acids at this site, including Isoleucine (I), Alanine (A), Tyrosine (Y), Leucine (L), Methionine (M) and G, but not S.

### Analyses of substitution rate

Substitution rates were estimated for synonymous and nonsynonymous sites within the 1365 bp *Or2 *sequences. The same data [Accession numbers: FJ606763] were collected from a closely related species of *C. solmsi*, *Ceratosolen cornutus*, which is the pollinator of *Ficus auriculata*. This addition increased the statistical power of the comparison of substitution rates [69]. Estimations of synonymous substitutions per synonymous site (*Ks*) between the dipteran species and *C. solmsi *ranged from 2.1785 to 3.1533 (Table 1). However, the estimates between the dipterans and *C. cornutus *were much lower, ranging from 1.3811 to 1.5638 only. In contrast, the rather similar estimates of substitutions occurred both between the dipterans and *P. pilosa *(1.1044–1.4665) and between the dipterans and *P*. sp. (1.0173–1.4974). Estimations of the number of nonsynonymous substitutions per nonsynonymous site (*Ka*) were slightly higher between the dipterans and *C. solmsi *(0.3064–0.3511) than between the dipterans and *C. cornutus *(0.2995–0.3373). The elevated substitution rate of the *Or2 *gene in *C. solmsi *also occurred when the analyses involved other hymenopterans (*N. vitripennis *and *Ap. mellifera*) instead of dipterans. Between-species comparisons in pollinators (*Ceratosolen*) yielded higher values of both *Ks *and *Ka *than in non-pollinators (*Philotrypesis*) (Table 1).

**Table 1:** Synonymous and nonsynonymous divergence of *Or2 *genes in fig wasps.

	*C. sm*	*C. c*	*A. bak*	*P. pil*	*P*. sp.	*N. vit*	*A. mel*	*A. aeg*	*C. qui*	*A. gam*	*D. mel*
*C. sm*		***0.0435***	0.0546	0.0610	0.0608	0.0499	0.1864	**0.3302**	**0.3064**	**0.3246**	**0.3511**
*C. c*	***0.6524***		0.0527	0.0514	0.0548	0.0462	0.1895	**0.3285**	**0.2995**	**0.3214**	**0.3373**
*A. bak*	1.3781	1.3191		0.0171	0.0181	0.0115	0.1755	**0.3272**	**0.3025**	**0.3219**	**0.3398**
*P. pil*	1.3686	1.1057	0.6505		***0.0090***	0.0146	0.1797	**0.3351**	**0.3094**	**0.3240**	**0.3443**
*P*. sp.	1.5050	1.0802	0.6121	***0.0577***		0.0156	0.1835	**0.3327**	**0.3043**	**0.3177**	**0.3416**
*N. vit*	1.5556	0.9242	0.6681	0.4286	0.3997		0.1847	0.3349	0.3075	0.3248	0.3386
*A. mel*	2.4788	1.6802	2.2714	1.7787	1.7499	1.9182		0.3173	0.3196	0.3265	0.3206
*A. aeg*	**2.1785**	**1.5638**	**1.7451**	**1.4665**	**1.4974**	1.2952	1.8865		0.0598	0.0802	0.1778
*C. qui*	**n.a**	**1.5110**	**1.6553**	**1.1044**	**1.0173**	0.8283	1.7399	0.7988		0.0750	0.1942
*A. gam*	**3.1533**	**1.3811**	**1.7594**	**1.2804**	**1.2362**	1.0298	1.5609	0.9125	0.6374		0.1737
*D. mel*	**2.5885**	**1.5277**	**1.4404**	**1.1202**	**1.0915**	0.9476	1.6724	1.1165	0.7818	0.9111	

Estimations of synonymous substitutions (*Ks*) according to Jukes and Cantor [135] are shown below the diagonal. The estimated nonsynonymous divergence (*K*a) according to Jukes and Cantor [135] are shown above the diagonal. n.a = not applicable. Estimates in bold correspond to synonymous and nonsynonymous divergence between species of Diptera and fig wasp groups. Estimates in bold Italic correspond to synonymous and nonsynonymous divergence between *C. solmsi *and *C. cornutus*, and between *P. pilosa *and *P*. sp. Species names are as follows: *C. sm, Ceratosolen solmsi*;*C. c, Ceratosolen cornutus*;*A. bak, Apocrypta bakeri*;*P. pil, Philotrypesis pilosa*; *P*. sp., *Philotrypesis *sp.;*N. vit, Nasonia vitripennis*;*A. mel, Apis mellifera*;*A. aeg, Aedes aegypti*;*C. qui, Culex quinquefasciatus*;*A. gam, Anopheles gambiae*;*D. mel, Drosophila melanogaster*.

Tajima's relative rate test [70] detected a significant difference in the rate of synonymous substitutions between *C. solmsi *and *C. cornutus *(Table 2). In contrast, these rates of substitutions were very similar between *P. pilosa *and *P*. sp. Significantly elevated substitution rates were not found when *C. cornutus *was compared with most of the NPFWs (except *P*. sp.). However, significantly elevated substitution rates were obtained when *C. solmsi *was compared with all NPFWs. No significant differences in the rate of nonsynonymous substitutions were detected between any lineages, although the number of substitutions at the 1st and 2nd codon positions was higher in *C. solmsi *than in *C. cornutus *and NPFWs.

**Table 2:** Results from Tajima's relative rate test for synonymous and nonsynonymous divergence of *Or2 *genes in fig wasps.

		Substitutions at the 3rd codon position			Substitutions at the 1st and 2nd codon positions		
		
Outgroup	SpeciesA-Species B	Ma	Mb	χ^2^	Na	Nb	χ^2^
*N. vit*	*P. pil- P*. sp.	13	7	1.80	3	4	0.14
*A. mel*	*A. bak- P. pil*	52	44	0.67	9	8	0.06
	*A. bak- P*. sp.	53	45	0.65	9	10	0.05
*D. mel*	*C. c- A. bak*	73	72	0.01	19	17	0.01
	*C. c- P. pil*	78	56	3.61	16	17	0.03
	*C. c- P*. sp.	79	56	3.92*	18	16	0.12
	*C. sm- C. c*	58	34	6.26*	20	12	2.00
	*C. sm- A. bak*	85	60	4.31*	22	12	2.94
	*C. sm- P. pil*	94	48	14.90***	22	15	1.32
	*C. sm- P*. sp.	99	52	14.63***	24	14	2.63

Ma and Mb are the numbers of substitutions at the 3^rd ^codon positions in the lineages leading to species A and B, respectively. Na and Nb are the number of substitutions at the 1st and 2nd codon positions in the lineages leading to species A and B, respectively. Species names are as in Table 1. *P *< 0.05 was used to reject the null hypothesis of equal rates between lineages. * 0.05 > *P *> 0.01; ** 0.01 > *P *> 0.001; *** *P *< 0.001.

The extent of the non-random substitutions at synonymous codons in different species was measured by three algorithms (Table 3). Congruent with the rate of synonymous substitutions (*Ks*) being inversely related to codon usage bias [71,72], *C. solmsi *showed the lowest codon bias. The NPFWs exhibited a much higher codon bias. C. *solmsi *had the lowest G+C content in their codons. Because the G+C content at second codon positions was similar in all species, the lower G+C content at (synonymous) third coding positions may have been the main cause of the lower codon bias of *C. solmsi*.

**Table 3:** Estimates of Codon Bias in *Or2 *genes of fig wasps.

Taxa	ENC	CBI	Scaled χ^2^	G+C2	G+C3s	G+Cc
Fig wasps associated with *F. hispida*						
*C. sm*	59.795	0.217	0.090	0.378	0.480	0.455
*A. bak*	55.213	0.306	0.178	0.376	0.588	0.493
*P. pil*	46.741	0.466	0.382	0.371	0.748	0.544
*P*. sp.	45.124	0.498	0.413	0.371	0.769	0.549
Other hymenopteran species						
*N. vit*	42.120	0.573	0.511	0.374	0.816	0.565
*A. mel*	58.591	0.236	0.123	0.367	0.594	0.483
Dipteran species						
*A. aeg*	50.176	0.347	0.309	0.370	0.656	0.511
*C. qui*	34.475	0.666	0.870	0.387	0.876	0.594
*A. gam*	42.589	0.508	0.472	0.391	0.768	0.557
*D. mel*	37.994	0.608	0.647	0.374	0.820	0.558

ENC, Effective Number of Codons. CBI, Codon Bias Index. SChi2, Scaled Chi Square. G+C2, G+C content at the second codon positions. G+C3s, G+C content at (synonymous) the third codon position; i.e. the G+C content in the third codon position excluding Trp and Met codons (nuclear universal genetic code). G+Cc, G+C content at codon positions. Species names are as in Table 1.

### Nucleotide diversity and neutrality tests for populations of fig wasps

Sequences from the N-terminal of the *Or2 *gene were collected from 20 individuals of each species in order to test the null hypotheses of strictly neutral evolution [73,74]. Following alignment, the 729 bp region revealed 18 haplotypes in *C. solmsi *[Accession numbers: FJ648225–FJ648242], 13 haplotypes in *A. bakeri *[Accession numbers: FJ648208, FJ648210, FJ648212–FJ648222], 15 haplotypes in *P. pilosa *[Accession numbers: FJ648244, FJ648246, FJ648250–FJ648262] and 11 haplotypes in *P*. sp. [Accession numbers: FJ648265, FJ648267, FJ648269, FJ648275–FJ648282]. Increased numbers of segregating sites were observed in *C. solmsi *(28 sites) rather than in NPFWs (21 sites in *A. bakeri*, 23 sites in *P. pilosa *and 20 sites in *P*. sp.). Assuming a randomly mating population at equilibrium, the average number of pairwise nucleotide differences between sequences (π) is expected to equal to average number of nucleotides segregating per site (θ). However, π was always smaller than θ (Table 4) and, thus, negative values of Tajima's *D *(*D*_*T*_) [74], Fu and Li's D* (D*) and F* (F*) [75] were observed for all populations of fig wasp.

**Table 4:** Neutrality tests for the *Or2 *gene of fig wasps using Tajima's *D*, Fu and Li's D* and F* statistics.

Sample	π (%)	θ (%)	*D*_*T*_	D*	F*
all sites					
*C. sm*	0.396	1.083	-2.473***	-3.700**	-3.883**
*A. bak*	0.349	0.812	-2.257**	-2.815*	-3.082**
*P. pil*	0.456	0.889	-1.872*	-2.401	-2.611*
*P*. sp.	0.357	0.773	-2.044*	-2.418	-2.683*
Nonsynonymous sites					
*C. sm*	0.451	1.218	-2.405**	-3.519**	-3.710**
*A. bak*	0.366	0.928	-2.251**	-2.989**	-3.221**
*P. pil*	0.460	1.044	-2.104*	-2.530*	-2.794*
*P*. sp.	0.474	0.986	-1.941*	-2.134	-2.413
Synonymous sites					
*C. sm*	0.288	0.812	-2.121*	-3.081**	-3.247**
*A. bak*	0.316	0.580	-1.739	-1.557	-1.857
*P. pil*	0.448	0.580	-0.694	-1.213	-1.232
*P*. sp.	0.123	0.348	-1.723	-2.386	-2.535

π, the average number of nucleotide differences per site between sequences; θ, average number of nucleotides segregating per site;*D*_*T*_, Tajima's *D *value; D*, Fu and Li's D* value; F*, Fu and Li's F* value. Species names are as in Table 1. * 0.05 > *P *> 0.02; ** 0.02 > *P *> 0.001; *** *P *< 0.001.

For all sites of N-terminal, the majority of tests were significantly less than zero, suggesting a departure from neutrality [74]. There were two notable exceptions to the departure from neutrality. D* tests in *P. pilosa *and *P*. sp. did not indicate significant departure from neutrality, although both the values were close to significance. However, both *D*_*T *_and F* tests for the two species of *Philotrypesis *were significantly less than zero. Similar to tests of neutrality for all sites, most of tests for nonsynonymous sites significantly rejected the model of strict neutrality, suggesting selective forces. Unlike neutrality test for all sites and nonsynonymous sites, none of the tests of synonymous sites in the NPFWs *Or2 *data was significant; these data were consistent with a neutral model of evolution. However, significantly negative *D*_*T*_, D* and F* tests were observed for synonymous sites of *Or2 *data in *C. solmsi*, indicating too many rare nucleotide polymorphisms with respect to predictions of the neutral theory [76].

## Discussion

### Identification of *Or2 *genes

We identified *Or83b *orthologs for the first time and from four species of fig wasps associated with *F. hispida*. As expected, their primary aa sequences with the *Or83b *subfamily were highly conserved relative to other insects [43,47-54]. They had > 60% identity and > 72% similarity, respectively, suggesting that the genes were orthologs in the different species. The extremely conservative C-terminal, especially at the last 164 aa, are involved in G-protein binding/activation required for downstream signal transduction [77]. The high identity and similarity of the aa sequences suggested that the four species of fig wasps are closely related species. This high degree of conservatism has not been observed in conventional ORs, even when compared to all 170 candidate ORs of *Ap. mellifera *[45], to 62 candidate ORs of *D. melanogaster *[44] and to 79 candidate ORs of *An. gambiae *[43]. This level of conservatism reflected a strong selective pressure on the aa sequence and was consistent with the important role of non-conventional receptors in the olfactory process [42]. The aa sequences of the four fig wasps included all the seven transmembrane domains of the G-protein coupled receptor. The group of threonines and tyrosines were extremely conserved (aa 465 and 466) in the region of TM 7 (Figure 1). They constituted candidate phosphorylation sites that may be important for regulating protein function [78]. An unusually long second intracellular loop was detected between TM 4 and TM 5. The function of this structural specialization remained unknown, even though the second extracellular loop of certain types of mammalian G protein-coupled receptors may be critical for ligand binding and affinity [79,80]. Recent bioinformatics and experimental investigations revealed that the membrane topology of ORs in *Drosophila *was, in fact, the inverse of mammalian GPCRs, with the N-terminal of these receptors located intracellularly [56,63]. Thus, we speculate that the long second intracellular loop of the non-conventional receptor in insects likely plays an important role in binding conventional ligand-binding ORs.

### Adaptation of spatial expression patterns of *Or2 *genes

We characterized the spatial expression patterns of *Or2 *genes in four species of fig wasp. RT-PCR experiments demonstrated that receptors were expressed only in adult male heads (including antennae and maxillary palps), but more widely in various tissues from adult females, including the antennae, legs and abdomens. The current view is that *Or83b *gene does not directly bind odorant ligands but rather it acts to form heterodimers with conventional ORs; this ensures appropriate dendritic localization and function [55-57]. The broad spatial expression patterns in OSNs further support the essential role of *Or83b *orthologs for a sense of smell [52,55]. Given the different habits of female and male fig wasps, the sex-tissue-specific expression of *Or2*-type receptors may indicate a role in host searching and oviposition in adult females.

Host plants are distributed patchily. Therefore, adult females must be able to distinguish the particular odours emitted by their host plant from the myriad of other volatile compounds. Such remarkable sensitivity and specificity is likely achieved by ORs expressed in the legs of adult female fig wasps. Likewise, *Or83b *orthologs are also expressed in legs of *An. gambiae *and *Ae. Aegypti*. Mosquito legs are known only as having a gustatory function [50,53]. Gustatory receptors (GRs) are the only chemosensory receptors whose expression has been detected in the legs of *D. melanogaster *[81,82], the location of gustatory sensilla. It seems likely that in addition to their olfactory function, *Or83b *orthologs in legs of fig wasps might function in a contact chemosensory pathway. Because they function in heterodimerization, perhaps *Or83b *orthologs are required in both olfactory and gustatory process. Chemosensory responses derived from legs would be extremely close to volatiles of fig fruit. These receptors in legs may play a critical role in evaluating the status of a host plant when fig wasps land for oviposition.

Lower levels of expression were detected in the abdomens of females in four species of fig wasp. Thus, the female's abdomen likely plays a critical role in locating an oviposition site. A small number of sensillae occur on the vaginal plate of the abdomen in *D. melanogaster*, suggesting a function in oviposition site selection in fruit flies [83]. In contrast to *Or2 *in the fig wasp, expression patterns of *Or83b *orthologs have not been discovered in the abdomens of other insects, including *D. melanogaster *(*DOr83b*) [47-49], *An. gambiae *(*AgOR7*) [50], *H. virescens *(*HvirR2*) [51], *Ap. mellifera *(*AmelR2*) [52] and *Ae. aegypti *(*AaOR7*) [53]. In the fig wasps, this pattern likely reflects an adaptive genetic change in response to their host. Both female PFWs and NPFWs use their ovipositor to touch fig inflorescences and for oviposition [37-39]. Chemosensory function in the abdomen of fig wasp may occur as described for the expression of *AgOR7 *in the proboscis and labellum of *An. gambiae *[50]. Abdominal chemoreception in all female fig wasps may function in both olfaction and gustation [84].

Support for the adaptation hypothesis is obtained from the males of all fig wasp species. Wingless male fig wasps usually live in the fig throughout their life. They do not need to search for a host. Mating is their primary task. Male fig wasps have highly specialized mouth parts for pulling females out of their galls, and, most importantly, for chewing an exit tunnel for newly transformed female wasps to leave the syconium [85-87]. In the male's enclosed environment, the broad expression of ORs may not be required. Thus, it is not surprising that female wasps express ORs in a greater number of tissues than do male wasps. In support of this hypothesis, few sensory hairs occur on a male's body.

We can not exclude the possibility that several conventional ORs independently express in some OSNs of other tissues, beside the head of males. *Or83b *orthologs do not always co-express with conventional ORs in all OSNs [48-50]. Indeed, the lack of *AgOR7 *(mosquito orthologs) expression in grooved peg sensillum of *An. gambiae *suggests the presence of an alternative pathway for olfactory signal transduction that is independent of *OR7 *function [50]. Unexpectedly, a weak band was obtained with cDNA prepared from the thorax of *C. solmsi*. Most NPFWs oviposit into ovaries of female flowers by inserting their ovipositor through the syconium wall (while not entering the syconium) [37-39]. Unlike NPFWs, female PFWs penetrate into the fig cavity through the ostiolar bracts and oviposit in the ovaries of the female flowers. In doing so, a PFW pollinates female flowers. Different oviposition behaviour of PFWs and NPFWs may cause different tissue-specific expression. In the process of entering the syconium's ostiole, the antennae of female PFWs are easily broken off. Expression of *Or2 *in the thorax and other tissues may help a pollinator to accurately locate oviposition sites in the dark inner syconium. PFWs seem to have less time to search for hosts because adult PFWs live for a much shorter period of time (from a few hours to 2 days) than do adult NPFWs (several days to 2 weeks) (personal observation). Thus, unlike NPFWs, PFWs may require a broad tissue expression of *Or2*, in part for increased sensitivity.

Tissue-specific gene expression implies that fig wasps may have cryptic olfactory inputs in tissues that express ORs. If the leg and/or abdomen of a fig wasp functions in olfaction, it is likely to be an exaptation [88]. The primary function of legs and the abdomen are movement and reproduction, respectively. The role of olfaction in these organs is a secondary function. The diversity of expression patterns may be important for determining species-specific olfactory profiles, such as in the detection of fruit odours by fruit flies [62] and human host odours by mosquitoes [50,53]. The patterns in fig wasps may reflect species-specific adaptations to ecology, habitat and physiology. Further study into the function and characteristics of *Or2 *and other conventional ORs will facilitate our understanding of co-evolution in this model system.

### Strong purifying selection for most orthologous amino acids

The evaluation of selection pressure used the *Ka/Ks *ratio, based on 19 orthologous *Or2 *genes. A very low average *Ka/Ks *ratio indicated that *Or2 *genes were mainly subjected to purifying pressure. This finding differed drastically from the properties of the ORs family, most often characterized by rapid evolution and highly species-specific gene repertoires [44,45,62]. A higher level of functional constraint on protein-coding exon sequences should lead to lower level of nonsynonymous variation which usually generates low average nonsynonymous:synonymous substitution ratios. Thus, the low average *Ka/Ks *ratio suggests that the *Or2*-type receptor plays an important role in the olfactory process of insects and is subject to a higher level of functional constraint. The 21st aa of *Or2 *appeared to be subjected to positive selection, although the *P*-value was not very significant. The aa in PFWs (S) differed from that in all NPFWs (G). Suzuki and Gojobori (1999) demonstrated that two amino acid sites of the human leukocyte antigen (HLA) gene undergoing positive selection might be involved in antigen recognition [89]. Other studies also showed that positive selection is focused mainly on the binding site and the distinct DNA-binding properties are determined by one or a few critical amino acids [90-94]. If the 21st aa performed a binding function, such divergence could have reflected ligand binding specificity between PFWs and NPFWs. Species-specific adaptive divergence might have been driven by the environment of the host [62]. Ecological niches differ between PFWs and NPFWs [37-39]. The composition and concentration of bouquets could vary between the inside and outside the syconium, although no data document this to occur. PFWs that enter the syconium are likely to smell extra syconian odours. Perhaps the change of aa at site 21 helped the *Or2 *of pollinators bind conventional ORs that respond to odours in the syconium.

### Elevated rate of substitution in pollinators

All PFWs and most NPFWs are currently classified as being part of the same chalcid family, Agaonidae [95-97]. However, recent molecular studies suggest that this family is paraphyletic and all PFWs form a monophyletic group [17,98]. An evaluation of closely related species should help to control for possible differences in mutation rate [69,99]. Thus, sequence data for *Or2 *genes were also collected from *C. cornutus*, a pollinator of *F. auriculata *and closely related to *C. solmsi*, to increase the power of our analysis of substitution rates.

Between-species comparisons of substitution rates involved two species of PFWs and three NPFWs. An elevated substitution rate was not detected in *Philotrypesis*, but it was in *C. solmsi*. The rapid evolution of *Or2 *genes in *C. solmsi *became more apparent when the closely related species in the genus *Ceratosolen *were compared. Low effective population sizes may be accompanied by relatively weak selection compared to drift yet usually drive an increase in the frequency of slightly deleterious substitutions [62,100-102]. However, no evidence suggests that PFWs have smaller population sizes than NPFWs. Typically, pollinators are almost always the dominant wasp (Herre EA & Machado CA, personal communication), though NPFWs might affect pollinator numbers by successfully invading the fig-pollinator mutualism system [103]. Thus, the hypothesis of a low effective population size can not be employed to explain these observations. Machado [104] found that species of *Ceratosolen *exhibited accelerated mutation in their mitochondrial genome relative to species in other genera. It is possible that this increased rate has affected the nuclear genome as well through cytonuclear associations [105-108]. For example, Rand et al. (2004) demonstrated that the association between nuclear and mitochondrial substitutions drives the evolutionary divergence [105]. Mishamar et al. (2006) showed that mitochondrial DNA and nuclear DNA complex I genes may have co-evolved [109].

Our results are consistent with the proposal that the rate of synonymous substitutions is inversely related to codon usage bias [71,72]. Codon usage bias was much lower in *C. solmsi *than in the NPFWs. The changed codon bias indicated that mutations were not strictly neutral [101,110]. Weak selection on synonymous mutations caused a codon usage bias in bacteria, yeast and flies [111-113]. In *Drosophila*, preferred codons also correspond to the more abundant tRNAs [114,115]. Because the most abundant tRNA translates their corresponding codon more rapidly, the preferred codon speeds up translation [116,117]. However, there are many counterexamples of highly expressed genes with little or no codon bias [118,119]. Therefore, we cannot unequivocally state that *Or2 *genes in PFWs that have lower codon usage bias are expressed at a lower level than in NPFWs. Conversely, the broader tissue expression of *Or2 *in PFWs than in NPFWs, together with the higher degree of host specificity of PFWs than NPFWS [13,18-23], implies that *Or2 *genes in PFWs are expected to be expressed at a higher level than in NPFWs.

### Non-neutral patterns of nucleotide variation

Olfaction genes of insects are widely assumed to experience selection [43,45,62,120-122]. Our investigation of *Or2 *polymorphism in four species of fig wasp sought evidence for selection, and the signature of selection can be detected by various tests [74,75,123]. These tests also tend to implicate the corresponding evolutionary forces. The significantly negative *D*_*T *_and F* values for *Or2 *data from populations of *C. solmsi, A. bakeri, P. pilosa *and *P*. sp. indicate a higher-than-expected number of low-frequency variations. This is consistent with purifying selection or directional selection reducing deleterious mutations in *Or2 *genes [74]. Once deleterious mutations appear in population, they will be maintained at a relatively low frequency due to selection pressures. And low-frequency variation will be increased more than in neutral conditions. For the DNA data, θ will be increased, and the test value will be significantly negative. With D*, *Or2 *data from *C. solmsi *and *A. bakeri *reject the neutrality hypothesis, while those from *P. pilosa *and *P*. sp. do not, although the values are close to significance (0.10 > *P *> 0.05)[75]. This result likely reflects either short length of sequence or a small sample size [124]. Increasing the length of the sequence and/or sample size could yield a more accurate result. Because both the *D*_*T *_and F* tests support a non-neutral model of evolution, most likely the *Or2 *data in *P. pilosa *and *P*. sp. also reflect selection.

Most tests on nonsynonymous sites of *Or2 *also show a significantly negative departure from neutrality, again suggesting possible purifying selection. Certainly, *Or2 *plays an important role in fig wasps and variations on nonsynonymous sites are maintained at a relatively low proportion. An alternative explanation for negative test values is the occurrence of directional selection. It eliminates deleterious mutations from the population and promotes the fixation of advantageous mutations that optimize function and lead to adaptation to an ecological niche. The *D*_*T *_test, together with D* and F* tests, clearly rejects the null hypothesis that the synonymoussites of *Or2 *gene in *C. solmsi *are evolving under a strictly neutral model of molecular evolution [74]. Selection pressures seem to also act against slightly deleterious mutations at synonymous sites within *C. solmsi*. Regarding synonymous sites in NPFWs, none of tests significantly rejected the null hypothesis. Thus, these data are consistent with a neutral model of evolution [74,75].

The use of several tests appears to be a more powerful means of inferring patterns of selection affecting nucleotide variance. Combined with test results on nonsynonymous sites, *Or2 *in *C. solmsi *appears to experience more effective selection relative to that of NPFWs. This might be related to the greater host specificity of PFWs relative to NPFWs, and/or it may also be related to the larger effective population size of PFWs. It remains to be determined whether this trend occurs just for *Or2 *or throughout the entire genome of *C. solmsi*. Data from additional loci are required to evaluate this possibility.

## Conclusion

*Or83b *orthologous genes obtained from one pollinator and three non-pollinator species of fig wasps associated with *F. hispida *were evaluated. We examined spatial expression patterns, evolutionary rate and selective forces acting on these genes in fig wasps.

*Or2 *genes were expressed beyond the known primary olfactory tissues and potentially this has functional implications [52]. *Or2 *plays an essential role in the localization and function of co-expressed OR proteins [55,57]. Thus, the expressions of *Or2 *genes in non-olfactory tissues of female fig wasps strongly indicated the presence of cryptic olfactory inputs in these tissues. Olfactory responses obtained from non-olfactory tissues may have indicated a long-term adaptation to figs. Future investigations can determine whether these genes have a chemosensory function expressed in non-olfactory tissues, or not.

Our results, taken together with those from previous studies [17], suggested that an accelerated rate of substitution was likely characteristic of the *Or2 *gene in *C.solmsi*, unlike that in NPFWs associated with *F. hispida*. Neutrality tests indicated that *Or2 *genes in most fig wasp populations were not concordant with the hypothesis of neutral selection. Considering the importance of *Or2*, selection against deleterious mutations could have maintained variations at a relatively low proportion. Unlike NPFWs, selection pressures were also detected in synonymous sites within *C.solmsi*, suggesting a more effective role on a pollinator species that is more specific to fig host.

## Methods

### Taxa sampling

Four species of fig wasp (*C. solmsi, A. bakeri, P. pilosa *and *P*. sp.) were randomly collected from fruits of *F. hispida *in Danzhou, Hainan province, China. The species, *C. cornutus *from *F. auriculata*, was collected for comparative analyses. Mature figs, from which the fig wasps emerge, were collected by Haoyuan Hu and Liming Niu, and dissected in the lab. All emerged fig wasps were immediately preserved in Sample Protector (TaKaRa). The four species were identified and then maintained at -80°C.

### Cloning the *Or83b *orthologous gene

Total RNA was isolated by using TRIzol procedures according to the manufacturer's instructions (Transgen Biotech). Each experiment used about 3 μg RNA for first-strand cDNA synthesis with EasyScript Reverse Transcriptase (Transgen Biotech) and Oligo(dT)_18 _primers (Invitrogen) to generate templates for individual PCR reactions. Seven degenerate primers were designed according to amino acid sequences retrieved from GenBank: *Nasonia vitripennis isoform 1 *[GenBank: XM_001607869], *Apis mellifera R2 *[GenBank: XM_001121145], *Drosophila melanogaster Or83b *[GenBank: NM079511], *Anopheles gambiae Or7 *[GenBank: AY363725], *Culex quinquefasciatus Or7 *[GenBank: DQ231246], *Bombyx mori R2 *[GenBank: AJ555487].

A short length of conserved C-terminal coding region (735 bp) was initially amplified using three degenerate primers: FW5'1: 5'-GYTNATHTTYGCNTGYGARC-3'; FW5'2: 5'-AAGGGCATCATGAAGCCCYTNATGGARYT-3'; FW3'1: 5'-TTACTTCAGCTGCACCARNACCATRAA-3'. FW5'2 and FW3'1 were designed using CODEHOP .

Based on the initial results, four additional degenerate primers were designed and used to obtain the remaining N-terminal coding region sequences: FW5'3: 5'-ATGATGAARWYNAAGCAWCARGG-3'; FW3'3: 5'-TTGCTRTADATNCCWCGNASRTC-3'; FW5'4: 5'ATGAARWYNAAGCAWCARGGNYTRRTNGCSGA-3'; FW3'4: 5'TGRTCNGCRCTKCCRGCCTTGAA-3'. A reaction volume of 50 μl contained 2 mM MgCl_2_, 0.2 mM of each dNTP, 0.2 μM of each primer, and 2.5 U of EasyTaq DNA polymerase (TransGen Biotech). PCR program involved 5 min at 94°C, then 35 cycles with 94°C for 30 s, 52°C for 40 s and 72°C for 1 min, followed by incubation for 10 min at 72°C. The amplified DNA products were purified and automated DNA sequencing was performed on an ABI3730 with an ABI PRISM BigDye terminator cycle sequencing ready reaction kit (Perkin-Elmer Biosystems). The same N-terminal data were obtained from 20 individuals per species of PFWs and NPFWs associated with *F. hispida*. These taxa were randomly collected from different trees and different crops. Thus, we were able to assess levels of intraspecific variation and test the neutrality model. Sequences from N-terminal and C-terminal were assembled as contiguous fragments using ContigExpress of Vector NTI Advance 9 (Invitrogen). The four full-length sequences of *Or83b *orthologous genes were obtained from four species of wasps associated with *F. hispida*. We also obtained a full-length sequence from *C. cornutus*. Thus, we were able to compare substitution rates in closely related species. The TMHMM v2.0  program [125,126] was used to predict the transmembrane domain (TM; Figure 1).

### Sequence alignment

Primary amino acid sequences of four species of fig wasp associated with *F. hispida *and 15 additional insect species were obtained from GenBank (accession numbers indicated in brackets): *Microplitis mediator Or1 *[EF141511], *Nasonia vitripennis isoform 1 *[XM_001607869], *Apis mellifera R2 *[XM_001121145], *Drosophila melanogaster Or83b *[NM079511], *Ceratitis capitata R2 *AY843206], *Anopheles gambiae Or7 *[AY363725], *Culex quinquefasciatus Or7 *[DQ231246], *Aedes aegypti Or7 *[AY582943], *Bombyx mori Or2 *[AJ555487], *Antheraea pernyi Or2 *[AJ555486], *Helicoverpa zea R2 *[AY843204], *Mamestra brassicae R2 *[AY485222], *Spodoptera exigua R2 *[AY862142], *S. litura R2 *[DQ845292] and *Tribolium castaneum R2 *[XM_968103]. Sequences were aligned by using CLUSTAL W [127] with default multiple alignment parameters. The alignment was optimized manually, and gap positions present in > 70% of the sequences were deleted. Sequence logos (Figure 1) were generated using Weblogo [128,129]. Both the identity and similarity values from all the possible comparisons were obtained using the EMBOSS Pairwise Alignment Algorithm .

### Phylogenetic analyses

Sequences having heterogeneous patterns of nucleotide or amino acid substitution may form erroneous branching patterns [130]. Therefore, we employed the Disparity Index Test [131] as implemented in MEGA 4.0 [132] to test 1st + 2nd codon positions, 3rd codon positions and the amino acid sequences for composition homogeneity among lineages. A Monte Carlo test (1000 replicates) was used to estimate the *P*-values [131]. A significantly heterogeneous pattern was detected at 3rd codon positions in most comparisons (*P *< 0.05). In contrast to 3rd codon position, the 1st + 2nd nucleotide positions and the amino acid sequences were found to be more homogeneous, except for 1st + 2nd codon position of *C. capitata *(data not show). Therefore, we used the 1st + 2nd codon positions and the amino acid sequences in the phylogenetic analysis. All positions containing gaps and missing data were eliminated from the dataset (complete deletion option). The final dataset here contained 864 aligned nucleotide positions and 430 aligned amino acid positions. The best tree was selected using the maximum parsimony (MP) criterion. Nodal stability was assessed using bootstrap proportions (1000 replicates) and the values are shown next to the branches. The MP trees were obtained using the Close-Neighbour-Interchange algorithm with search level 3, in which the initial trees were obtained with the random addition of sequences (10 replicates). Phylogenetic analyses were conducted in MEGA 4.0 [132] and PAUP* 4.0b10 [64].

### RNA expression

Antennae, thoraxes, abdomens, legs and heads (male) of four species of fig wasp associated with *F. hispida *were dissected in Sample Protector (TaKaRa), and total RNA of each tissue was isolated by using TRIzol procedures according to the manufacturer's instructions (Transgen Biotech). A series of non-quantitative RT-PCR experiments were performed by using cDNA preparations from various tissues of all male or female fig wasps and the same primer pairs were used for each type of tissue. In order to control for genomic DNA contamination, primers spanning predicted introns were designed for subsequence NEST-PCR reactions: FW5'5: 5'-AGTGCBATCAARTAYTGGGTNGA-3'; FW5'6: 5'-CTNGCNTACCARGCNAC NAA-3'; FW3'5: 5'-TTACTTCAGCTGCACCARN ACCATRAA-3'. All RT-PCR reactions were replicated at least three times. We also amplified the *actin *gene [133] from each tissue as a control for cDNA integrity by use of the following primers: β-*actin *F: 5'-ATGTGCAAGGCHGGHTTCGC-3'; β-*actin *R: 5'-CRTGGATRCCGCA VGAYTCC-3'. PCR products were purified and directly sequenced as described above.

### Selective pressure on individual codons

Selective pressure (positive selection and purifying selection) on individual codons (sites) within the coding region of the 19 amino acid sequences of *Or83b *orthologs were inferred using the Single Likelihood Ancestor Counting (SLAC) package [89]. This codon-based maximum likelihood method did not assume equal synonymous substitution rates throughout the sequence and it chose the most appropriate model for nucleotide substitution.

### Substitution rate

Evolutionary divergence at the gene-coding region was estimated by the number of synonymous substitutions per synonymous site (*Ks*) and the number of nonsynonymous substitutions per nonsynonymous site (*Ka*) [68]. Analyses were conducted using the modified Nei-Gojobori (Jukes-Cantor) method (assumed transition/transversion bias = 1.021) in MEGA4 [132,134,135]. The results were based on the sequences alignment of seven hymenopteran species (*C. solmsi, C. cornutus*, *A. bakeri, P. pilosa, P*. sp., *N. vitripennisl *and *Ap. mellifera*) and four dipteran species (*Ae. aegypti, C. quinquefasciatus, An. gambiae *and *D. melanogaster*). All positions containing alignment gaps and missing data were eliminated only in pairwise sequence comparisons (pairwise deletion option).

Equality of evolutionary rate between two lineages of fig wasps was tested using an outgroup in Tajima's relative rate test in MEGA4 [70,132].*P *< 0.05 was used to reject the null hypothesis of equal rates between lineages.

Effective Number of Codons (ENC) [136], Codon Bias Index (CBI) [137] and Scaled Chi-square (SChi2) [113] methods were implemented in DnaSP program version 4.50.3 [138]. These methods estimated the codon bias present at the gene-coding region. The value of ENC ranges from 20 (only one codon is used for each amino acid; i.e., the codon bias is maximum) to 61 (all synonymous codons for each amino acid are equally used; i.e., no codon bias). CBI values range from 0 (uniform use of synonymous codons) to 1 (maximum codon bias). SChi2 measured the difference between the observed number of codons and those expected from equal usage of codons. A higher SChi2 value indicated a stronger deviation from the random use of synonymous codons.

### Neutrality test

The signature of selection can be detected by various tests [74,75,123], but it is not clear which is most powerful. Thus, we exploited Tajima's *D *statistic [74], and Fu and Li's D* and F* tests [75] to estimate deviations from neutral expectations. These tests were implemented in DnaSP version 4.50.3 [138]. A negative Tajima's *D *signified an excess of low frequency polymorphisms, indicating population size expansion, purifying selection, recent directional selection and/or background selection of deleterious mutation. A positive Tajima's *D *signified low levels of both low and high frequency polymorphisms, indicating a decrease in population size and/or balancing selection [74]. The same thing held for Fu and Li's tests.

## Authors' contributions

BL carried out the majority of the molecular work, designed and conceived the study and drafted the manuscript. NW designed some primers and carried out a portion of the molecular work. JX participated in the design of the study. YX coordinated the study. RWM contributed to data analysis, interpretations of phylogenetic inference and participated in the writing. DH participated in the design of the study, directed the research and helped to draft the manuscript. All authors read and approved the final manuscript.
